# One sixth of primary anterior cruciate ligament reconstructions may undergo reoperation due to complications or new injuries within 2 years

**DOI:** 10.1007/s00167-020-06127-w

**Published:** 2020-06-29

**Authors:** Lise Lord, Riccardo Cristiani, Gunnar Edman, Magnus Forssblad, Anders Stålman

**Affiliations:** 1 Department of Molecular Medicine and Surgery, Stockholm Sports Trauma Research Center, Karolinska Institutet, Stockholm, Sweden; 2 Capio Artro Clinic, FIFA Medical Centre of Excellence, Sophiahemmet Hospital, Valhallavägen 91, 11486 Stockholm, Sweden

**Keywords:** Anterior cruciate ligament, ACL, ACL reconstruction, ACLR, Reoperation, Revision ACL

## Abstract

**Purpose:**

To analyse the incidence, types and risk factors for reoperation within 2 years of primary anterior cruciate ligament reconstruction (ACLR).

**Methods:**

Our clinic registry was used to identify primary ACLRs, performed from 2005 to 2015, and reoperations performed on the ipsilateral knee within 2 years at our institution. Reoperations were identified using procedural codes and analysis of medical records. A logistic regression analysis was used to evaluate risk factors for reoperation.

**Results:**

A total of 6030 primary ACLRs were included. A total of 1112 (18.4%) reoperations performed on 1018 (16.9%) primary ACLRs were identified. The most common reoperations were screw removal (*n* = 282, 4.7%), meniscus procedures (*n* = 238, 3.9%), cyclops removal/notchplasty (*n* = 222, 3.7%) and reoperations due to graft rupture (*n* = 146, 2.4%), including revision ACLR. Age < 30 years (OR 1.57; 95% CI 1.37–1.80; *P* < 0.001), female gender (OR 1.33; 95% CI 1.17–1.51; *P* < 0.001), medial meniscus repair (OR 1.55; 95% CI 1.23–1.97; *P* < 0.001), lateral meniscus resection (OR 1.26; 95% CI 1.07–1.49; *P* = 0.005) and lateral meniscus repair (OR 1.38; 95% CI 1.03–1.85; *P* = 0.02) at primary ACLR were found to be risk factors for reoperation.

**Conclusion:**

One sixth of all primary ACLRs underwent reoperation due to complications or new injuries within 2 years. The most common reoperations were screw removal, meniscus procedures, cyclops removal/notchplasty and reoperations due to graft rupture, including revision ACLR. Younger age (< 30 years), female gender, medial meniscus repair and lateral meniscus resection or repair at primary ACLR were associated with an increased risk of reoperation. This study provides clinicians with important data to inform patients about the short-term reoperation rates, the most common reoperation procedures and risk factors for reoperation after primary ACLR.

**Level of evidence:**

III.

## Introduction

Anterior cruciate ligament (ACL) tears are common and are often treated with surgical reconstruction to restore joint laxity and minimise the risk of injuries to other joint structures [6, 11]. If concomitant ligament, meniscus or cartilage injuries are present, they are often addressed at the same operation [6]. An ACL injury is a serious knee injury with a great risk of persistent morbidity and need for further surgery [9, 14]. Few studies have investigated the incidence, types and risk factors for reoperation after ACL reconstruction (ACLR). Conflicting results can be found in the literature, with studies reporting reoperation rates between 6.5 and 34%, depending on the types of reoperation studied and the length of follow-up [9, 14]. The most commonly studied reoperation is revision ACLR, with an incidence reported to be between 3.6 and 7% after primary ACLR [12–14, 18]. Other less studied reoperations are meniscus procedures, cartilage debridement or microfracture, hardware removal and procedures due to joint stiffness, such as extension deficits [5, 10]. Proposed risk factors for reoperation are multifactorial and include younger age and meniscus or cartilage injuries at the time of primary ACLR [5].

To date, questions remain about the incidence, types and risk factors for reoperation after primary ACLR.

The purpose of this study was to analyse the incidence, types and risk factors for reoperation within 2 years of primary ACLR, in a large cohort. It was hypothesised that continued problems, which lead to reoperations, are relatively common after ACLR and that younger age, female gender and meniscus repair at the time of primary ACLR are risk factors for reoperation.

## Materials and methods

Ethical approval for this study was obtained from the regional ethics committee, Karolinska Institutet (Diarie-number 2016/1613-31/32).

Data were extracted from our clinic registry. Primary ACLRs, performed from 2005 to 2015, and reoperations performed within 2 years on the ipsilateral knee were identified. First, all primary ACLRs were identified. If a patient underwent bilateral ACLR, each knee was considered a separate case. Patients’ characteristics at the time of primary ACLR were reviewed. The data collected were age, gender, side of operation, graft type, fixation methods, cartilage and meniscus injuries and meniscus resection or repair. Reoperations performed within 2 years of primary ACLR were then identified. Surgical procedures were coded according to the NOMESCO (Nordic Medico-Statistical Committee) classification of surgical procedures [16]. If a reoperation had a specific procedural code, such as NGE41 (anterior cruciate ligament reconstruction), NGD11 (partial meniscectomy), NGD21 (meniscal repair), or NGU49 (hardware removal), no analysis of medical records was performed. If a more general code was used, such as NGA11 (diagnostic arthroscopy) or NGF31 (arthroscopic debridement of knee joint), medical records were reviewed to identify the exact type of reoperation. A reoperation and a non-reoperation cohort were, therefore, established.

The reoperation cohort was divided into subgroups depending on the procedure performed. Eleven reoperation subgroups were identified: screw removal, meniscus procedures (resection or repair), cartilage procedures (microfracture, debridement, abrasion), cyclops removal/ notchplasty, septic arthritis (lavage, debridement), graft rupture (including patients who underwent arthroscopy confirming graft rupture and revision ACLR without previous arthroscopy), synovitis (shaving of synovia), arthrofibrosis (arthroscopy due to joint stiffness and scar tissue formation), diagnostic arthroscopy without a clear diagnosis, removal of loose bodies and others (excision of osteophytes, ganglion or bone, as well as tibia osteotomy and scar correction). For patients who underwent multiple reoperations for the same reason, such as septic arthritis that led to multiple debridements, only the first reoperation was included in the analysis. If one patient underwent multiple reoperations or medical records showed that multiple procedures were performed during one reoperation, the patient was included in each reoperation subgroup that matched each procedure performed. One patient could, therefore, be present in multiple reoperation subgroups.

### Surgical technique and rehabilitation of primary anterior cruciate ligament reconstruction

All patients underwent surgery using a single-bundle autologous hamstring tendon (HT) or bone–patellar tendon–bone (BPTB) technique. For the ACLRs performed with HT grafts, the semitendinosus tendon was primarily harvested and prepared as a triple or quadruple graft. If the length or the diameter of the graft was considered insufficient (< 8 mm), the gracilis tendon was additionally harvested and combined with the semitendinosus graft. The BPTB graft was harvested as the central third of the patellar tendon with two bone blocks. The femoral tunnel was drilled using an anteromedial portal technique. Both grafts were routinely fixed using an Endobutton fixation device (Smith and Nephew, Andover, Mass, USA) on the femoral side and Ethibond no. 2 sutures (Ethicon, Sommerville, NJ) tied over a 4.5-mm AO bicortical screw with a washer (Smith and Nephew, Andover, Mass, USA) as a post or an interference screw on the tibial side. A minority of the cases were fixed using other methods, such as a RigidFix Cross Pin device (DePuy Mitek, Raynham, MA) for femoral fixation, an Intrafix device (DePuy, Mitek, Raynham, MA) for tibial fixation, or interference screws for femoral or tibial fixation. Meniscal repair was performed for both the medial meniscus and lateral meniscus with an arthroscopic all-inside technique using the FastFix suture anchor device (Smith & Nephew, Andover, Mass, USA) for tears located in the dorsal or middle portion. An outside-in technique, using PDS 0 (Ethicon, Sommerville, NJ), was performed for tears in the anterior portion of the meniscus. All the patients followed a standardised rehabilitation protocol. In the event of an isolated ACLR or ACLR with simultaneous meniscal resection, full weight bearing and full range of motion were encouraged as tolerated.

If meniscal repair was performed, patients wore a hinged knee brace for 6 weeks. Flexion was limited from 0° to 30° for the first 2 weeks, from 0° to 60° for the third and fourth weeks and from 0° to 90° for the fifth and sixth weeks after surgery. For all patients, quadriceps strengthening was restricted to closed kinetic chain exercises during the first 3 months. On the basis of muscle strength, co-ordination and functional performance, the patients were allowed to return to sports 6 months postoperatively at the earliest.

### Statistical analysis

All the data were analysed using the Statistical Package for Social Sciences, SPSS (Version 24.0 Armonk, IBM Corp., New York, USA). Demographic data were summarised using descriptive statistics such as frequency, mean and standard deviations. Comparisons of patients’ characteristics between the non-reoperation and reoperation cohort and between the non-reoperation cohort and reoperation subgroups were performed with an independent Student’s *t* test for continuous variables and Pearson’s chi-square test for categorical variables. A logistic regression analysis was performed with age, gender, cartilage injury, medial meniscus resection, medial meniscus repair and lateral meniscus resection, or lateral meniscus repair at the time of primary ACLR as independent variables and reoperation as the dependent variable. Age was dichotomised into classes close to the median (< 30 years vs. ≥ 30 years). The results of the logistic regression analysis were expressed as odds ratios (OR) with 95% confidence intervals (CI). The level of significance in all analyses was 5% (two tailed).

## Results

**Fig. 1:**
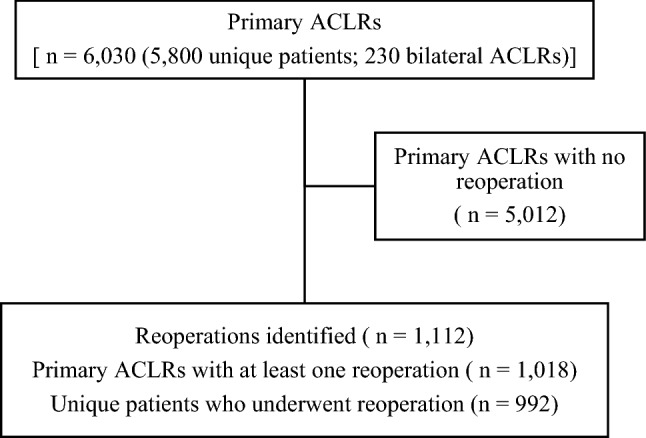
Patient flowchart. *ACLR* anterior cruciate ligament reconstruction

A total of 6030 primary ACLRs (5800 unique patients; 230 bilateral ACLRs) were included. A total of 1112 (18.4%) reoperations performed within 2 years on 1018 (16.9%) primary ACLRs were identified. A number of 992 unique patients underwent reoperation (Fig. 1). The mean (SD) time from primary ACLR to reoperation was 11.7 (5.8) months.

### Comparison between reoperation and non-reoperation cohort

Patient characteristics at primary ACLR and a comparison between the non-reoperation and reoperation cohort are summarised in Table 1. The mean age at primary ACLR was significantly lower (*P* < 0.001) for the reoperation cohort, although the difference was only 2.6 years. Moreover, females were represented to a significantly higher degree in the reoperation cohort (51.6% vs. 43.0%; *P* < 0.001). Differences between the cohorts were also present with regard to the use of femoral (*P* = 0.001) and tibial (*P* = 0.02) fixation devices. The use of an Endobutton (Smith & Nephew, Andover, MA) (87.5% vs. 82.7%) for femoral fixation and an AO bicortical screw with a washer (Smith & Nephew, Andover, MA) (79.3% vs. 74.9%) for tibial fixation was more common in the reoperation cohort. The reoperation cohort had a significantly larger number of meniscus injuries (*P* = 0.002) and underwent a significantly larger number of medial meniscus repairs (*P* < 0.001) and lateral meniscus repairs (*P* = 0.003) at primary ACLR. Meanwhile, no significant differences were found between the cohorts with regard to the other variables (Table 1). Types of reoperation are detailed in Table 2.

**Table 1:** Patient characteristics

	Primary ACLR	Non-reoperation cohort	Reoperation cohort	*P *value*
Number	6030	5012	1018	
Age at primary ACLR, years, mean ± SD	28.3 ± 10.7	28.7 ± 10.7	26.1 ± 10.2	< 0.001
Gender				< 0.001
Male	55.5	57.0	48.4	
Female	44.5	43.0	51.6	
Side				n.s
Left	48.6	48.9	47.3	
Right	51.4	51.1	52.7	
Graft type				n.s
HT autograft	93.8	93.7	94.3	
BPTB autograft	6.2	6.3	5.7	
Femoral fixation				0.001
Endobutton	83.5	82.7	87.5	
Rigidfix	11.8	12.5	8.4	
Interference screw	4.6	4.7	4.1	
Other	< 0.1	0.1	0	
Tibial fixation				0.02
AO screw	75.6	74.9	79.3	
Intrafix	6.3	6.6	4.9	
Interference screw	12.1	12.3	10.9	
Other	6.0	6.2	4.9	
Cartilage injury	18.2	18.5	17.0	n.s
Meniscus injury	47.4	46.4	52.3	0.002
Medial meniscus	23.8	23.6	24.9	
Lateral meniscus	23.6	22.8	27.4	
Meniscus surgery				
Medial meniscus resection	14.8	15.2	13.1	n.s
Medial meniscus repair	6.1	5.4	9.2	< 0.001
Lateral meniscus resection	16.2	15.8	18.2	n.s
Lateral meniscus repair	3.9	3.6	5.6	0.003

Data are reported as %, unless otherwise indicated

*ACLR* anterior cruciate ligament reconstruction, *BPTB* bone–patellar tendon–bone, *HT* hamstring tendons, *SD* standard deviation

*P values for comparisons between the non-reoperation and reoperation cohort

**Table 2:** Types of reoperation

Reoperation	Number	% of all primary ACLRs	% of reoperations
Screw removal	282	4.7	25.3
Meniscus procedures	238	3.9	21.4
Cyclops removal/notchplasty	222	3.7	20.0
Graft rupture	146	2.4	13.1
Cartilage procedures	77	1.3	6.9
Septic arthritis	53	0.9	4.8
Synovitis	31	0.5	2.8
Diagnostic arthroscopy	22	0.4	2.0
Extraction of loose body	16	0.3	1.4
Arthrofibrosis	16	0.3	1.4
Other*	9	0.2	0.8
Total	1112		

The “revision ACLR” subgroup (*n* = 134) is included in the “graft rupture” subgroup and, therefore, already included in the overall sum

*ACLR* anterior cruciate ligament reconstruction

*Includes: excision of osteophytes, ganglion or bone, tibial osteotomy, scar correction

### Comparison between reoperation subgroups and non-reoperation cohort

Patient characteristics at the time of primary ACLR for each reoperation subgroup are detailed in Table 3. The reported *P* values compare the reoperation subgroup with the non-reoperation cohort. The mean age of the “screw removal”, “meniscus procedures”, “cyclops removal/notchplasty” and “graft rupture” subgroups was significantly lower; whereas, the mean age of the “cartilage procedures” subgroup was significantly higher than the mean age of the non-reoperation cohort. Females were represented to a significantly higher degree in the “screw removal” and “cyclops removal/notchplasty” subgroups compared with the non-reoperation cohort. A larger number of HT autografts were present in the “screw removal” subgroup; whereas, a larger number of BPTB autografts were present in the “cyclops removal/notchplasty” subgroup in comparison with the non-reoperation cohort. A larger number of Endobutton (Smith & Nephew, Andover, MA) and a smaller number of RigidFix (DePuy Mitek, Raynham, MA) fixation devices for femoral fixation were found in the “graft rupture” subgroup in comparison with the non-reoperation cohort. A significantly larger number of AO bicortical screws with a washer (Smith & Nephew, Andover, MA) for tibial fixation were found in the “screw removal” subgroup. A total of 5.3% of patients who underwent ACLR using an AO bicortical screw for tibial fixation underwent screw removal at follow-up, in comparison with 2.7% of the patients who underwent ACLR using other types of tibial fixation (data not shown in Table 3). The number of meniscal injuries, for both the medial and lateral meniscus, at the time of primary ACLR was significantly higher in the “meniscus procedure” reoperation subgroups. In particular, this subgroup underwent a significantly larger number of medial meniscus repairs, lateral meniscus resections and lateral meniscus repairs at the time of primary ACLR in comparison with the non-reoperation cohort. The reoperation rate within 2 years was 14.4% for medial meniscus repair, 10% for lateral meniscus repair and 4.3% for lateral meniscus resection performed in conjunction with primary ACLR (data not shown in Table 3). Finally, a larger number of lateral meniscus resections and cartilage injuries at the time of primary ACLR were present in the “cartilage procedure” reoperation subgroup in comparison with the non-reoperation cohort (Table 3).

**Table 3:** Patient characteristics at the time of primary ACLR for each reoperation subgroup

	Screw removal	*P* value	Meniscus procedures	*P* value	Cyclops removal/notchplasty	*P* value	Graft rupture	*P* value	Cartilage procedures	*P* value	Septic arthritis	*P* value
Number (% of primary ACLRs)	282 (4.7)		238 (3.9)		222 (3.7)		146 (2.4)		77 (1.3)		53 (0.9)	
Mean time interval from primary ACLR to reoperation, months ± SD	12.7 ± 4.6		13.0 ± 5.4		10.5 ± 4.9		13.7 ± 5.2		14.5 ± 6.6		0.2 ± 0.8	
Age at primary ACLR, years, mean ± SD	27.4 ± 10.5	0.04	24.0 ± 9.7	< 0.001	26.4 ± 9.5	0.001	21.4 ± 7.8	< 0.001	31.5 ± 9.9	0.02	28.9 ± 9.9	n.s
Gender		< 0.001		n.s		< 0.001		n.s		n.s		n.s
Male	37.2		54.2		44.6		53.7		67.5		62.3	
Female	62.8		45.8		55.4		46.3		32.5		37.7	
Side		n.s		n.s		n.s		n.s		n.s		n.s
Left	49.6		50.0		42.3		52.7		48.1		35.8	
Right	50.4		50.0		57.7		47.3		51.9		64.2	
Graft type		0.02		n.s		0.03		n.s		n.s		n.s
HT autograft	97.2		93.3		90.1		97.3		88.3		100	
BPTB autograft	2.8		6.7		9.9		2.7		11.7		0	
Femoral fixation		0.002		n.s		n.s		0.01		n.s		n.s
Rigidfix	5.7		7.6		11.3		5.5		11.7		9.4	
Endobutton	91.5		86.6		84.7		93.2		83.1		90.6	
Interference screw	2.8		5.8		4.0		1.3		5.2		0	
Other	0		0		0		0		0		0	
Tibial fixation		0.001		n.s		n.s		n.s		n.s		0.01
AO screw	85.8		75.6		77.0		84.2		71.4		86.8	
Intrafix	3.9		4.2		6.3		3.4		3.9		11.3	
Interference screw	6.7		16.0		12.2		8.2		13.0		0	
Other	3.5		4.2		4.5		4.0		11.7		1.9	
Cartilage injury	15.6	n.s	16.4	n.s	13.5	n.s	11.0	0.02	35.1	< 0.001	24.5	n.s
Meniscus injury												
Medial meniscus	19.9	n.s	37.8	< 0.001	22.5	n.s	16.4	0.04	26.0	n.s	26.4	n.s
Lateral meniscus	24.8	n.s	36.6	< 0.001	25.7	n.s	26.7	n.s	33.8	0.02	24.5	n.s
Meniscus surgery												
MM resection	12.4	n.s	11.3	n.s	11.3	n.s	11.0	n.s	20.8	n.s	20.8	n.s
MM repair	5.7	n.s	22.3	< 0.001	9.0	0.02	4.1	n.s	3.9	n.s	3.8	n.s
LM resection	17.0	n.s	22.7	0.004	17.6	n.s	17.8	n.s	28.6	0.004	13.2	n.s
LM repair	2.8	n.s	10.5	< 0.001	5.0	n.s	6.2	n.s	3.9	n.s	5.7	n.s

Data are reported as %, unless otherwise indicated. The “revision ACLR” subgroup (*n* = 134; 2.2%) is included in the “graft rupture” subgroup and, therefore, not separately shown in the table. *P *values are for comparisons between the reoperation subgroup and the non-reoperation cohort (patient characteristics of the non-reoperation cohort are detailed in Table 1)

*ACLR* anterior cruciate ligament reconstruction, *BPTB* bone–patellar tendon–bone, *HT* hamstring tendon, *LM* lateral meniscus, *MM* medial meniscus, *SD* standard deviation

### Analysis of risk factors for reoperation

Logistic regression analysis revealed that the risk of undergoing a reoperation was significantly related to younger age (< 30 years), female gender, medial meniscus repair and lateral meniscus resection or repair at the time of primary ACLR. Medial meniscus resection or the presence of a cartilage injury at the time of primary ACLR were not associated with an increased risk of reoperation within 2 years (Table 4).

**Table 4:** Risk factors for reoperation after primary ACLR in logistic regression analysis

Risk factor	Regression coefficient (*ß*)	S.E	OR (95% CI)	*P *value
Age < 30 years	0.45	0.07	1.57 (1.37–1.80)	< 0.001
Female gender	0.28	0.06	1.33 (1.17–1.51)	< 0.001
Cartilage injury	0.02	0.08	1.03 (0.86–1.22)	n.s
Medial meniscus resection	− 0.05	0.09	0.94 (0.78–1.14)	n.s
Medial meniscus repair	0.44	0.12	1.55 (1.23–1.97)	< 0.001
Lateral meniscus resection	0.23	0.08	1.26 (1.07–1.49)	0.005
Lateral meniscus repair	0.32	0.14	1.38 (1.03–1.85)	0.02

*ACLR* anterior cruciate ligament reconstruction, *CI* confidence intervals, *OR* odds ratio, *S.E* standard error

## Discussion

The most important finding in the present study was that about one sixth of all primary ACLRs underwent a reoperation within 2 years due to persistent problems or new injuries. The most common reoperations were screw removal, meniscus procedures, cyclops removal/notchplasty, reoperations due to graft rupture (including revision ACLR) and cartilage procedures. Age < 30 years, female gender, medial meniscus repair and lateral meniscus resection or repair at the time of primary ACLR were found to be independent risk factors for reoperation.

Conflicting results are reported in the literature regarding the rate of reoperation after primary ACLR. The reoperation rate found in the present study was higher than that reported by Csintalan et al. [5], who found a reoperation rate of 3.9% at a mean follow-up of 1.9 years. Lyman et al. [14] reported a reoperation rate of 6.5% at the 1-year follow-up. Van Dijck et al. [7] followed patients for 7.4 years after primary ACLR and found a reoperation rate of 27.6%; whereas, Hettrich et al. [10] found a reoperation rate of 18.9% at the 6-year follow-up.

What may in part explain the different reoperation rates between our study and the study by Csintalan et al. [5] is that, in our 2-year follow-up, we included all reoperations performed on a single primary ACLR. This ensured that eventual additional reoperations were included. On the other hand, Csintalan et al. [5] only included the four most common reoperation procedures (cartilage and meniscus procedures, hardware removal and procedures due to joint stiffness) in their study and their follow-up time was limited to the occurrence of the first reoperation.

The most common reoperation in the present study was screw removal (*n* = 282, 4.7%). In most cases, the reason for screw removal is pain over the proximal tibia. Previous studies have reported hardware removal in a range of between 0.6 and 0.7% and 12.7% of primary ACLRs [5, 7, 20]. Differences regarding the incidence of hardware removal may be due to different surgical techniques. In the present study, a bicortical AO screw with a washer as a post was used for tibial fixation in 75.6% of primary ACLRs. The AO screw could be responsible for local pain and discomfort. The removal of an AO screw can be easily performed under local anaesthesia and with a small incision. This may also explain the higher reoperation rate found when this type of tibial fixation was used. Surgeons could be more prone to suggest a reoperation for screw removal, due to the relative ease of the procedure. In the present study, among all patients who underwent ACLR using an AO screw for tibial fixation, 5.3% underwent screw removal, in comparison with 2.7% of the patients who underwent ACLR using other types of tibial fixation.

The second most common reoperation (*n* = 238, 3.9%) was meniscus procedures. This reoperation subgroup underwent a significantly larger number of medial meniscus repairs, lateral meniscus resections and lateral meniscus repairs at the time of primary ACLR in comparison with the non-reoperation cohort. Medial meniscus repair and lateral meniscus resection or repair were also found to be significant risk factors for reoperation in our logistic regression analysis. The reoperation rate at 2 years for medial meniscus and lateral meniscus repair was 14.4% and 10%, respectively. These reoperation rates are in line with those reported in previous studies [3, 15, 17, 19]. The medial meniscus resists anterior tibial translation in the ACL-reconstructed knee [2, 4]. This may put the repaired medial meniscus under greater stress, contributing to more failures and subsequent reoperations.

The third most common reoperation was cyclops removal/notchplasty (*n* = 222, 3.7%). In a previous study, Wang et al. [22] found that only about 13% of the cyclops lesions caused extension deficits after primary ACLR. In the present study, it is unclear how many patients had an extension deficit as a primary indication for surgery and how many had joint pain which led to a diagnostic arthroscopy where a cyclops removal/notchplasty was performed and registered as a reoperation. It is also possible that the indication for surgery was pain due to meniscus or cartilage problems and, when a cyclops removal/notchplasty was performed at the same time, this was also registered as a reoperation. Moreover, with this study, we are unable to say whether the surgery performed was a cyclops removal or notchplasty or a combination of these procedures. A larger number of primary ACLRs performed with a BPTB autograft were present in the “cyclops removal/notchplasty” reoperation subgroup in comparison with the non-reoperation cohort. However, the BPTB graft group was much smaller than the HT group, comprising only 6.2% of all primary ACLRs. It is, therefore, difficult to draw conclusions from this finding.

The fourth most common reoperation was due to graft rupture (*n* = 146, 2.4%). Of these, 134 (2.2%) underwent revision ACLR. The other 12 cases, which underwent a diagnostic arthroscopy, were subsequently treated with rehabilitation or revision ACLR was performed more than 2 years after primary ACLR. A larger number of Endobutton (Smith & Nephew, Andover, MA) and a smaller number of RigidFix (DePuy Mitek, Raynham, MA) fixation devices for femoral fixation were found in the “graft rupture” reoperation subgroup compared with the non-reoperation cohort. A previous study from the Danish ACL reconstruction registry, comparing ACL graft fixation methods using an HT graft, found that cortical suspensory fixation is associated with an increased risk of revision; whereas, intra-tunnel transfixation is associated with a reduced risk of revision ACLR [8].

Age < 30 years at the time of primary ACLR was found to be a significant risk factor for reoperation. The mean age of the “screw removal”, “meniscus procedures”, “cyclops removal/notchplasty” and “graft rupture” (including revision ACLR) reoperation subgroups was significantly lower compared with the non-reoperation cohort. Younger patients are more likely to return to a high level of activity and contact sports than older patients. As a result, they may have more injuries and require further surgery more often after ACLR. Previous studies have reported in detail the association between younger age and a higher risk of graft rupture and revision ACLR [1, 21].

In the present study, female gender was also found to be a risk factor for reoperation after primary ACLR. Females were represented to a significantly higher degree in the “screw removal” and “cyclops removal/notchplasty” reoperation subgroups than the non-reoperation cohort.

The main strength of this study was the analysis of a large cohort. This enabled a comprehensive analysis of incidence, types and risk factors for reoperation. In addition, all the patients underwent surgery at the same institution.

There are several limitations. First is the limited follow-up of 2 years after primary ACLR. There is probably a higher reoperation rate at a longer follow-up. Another limitation is that the study only comprised patients who underwent both primary ACLR and reoperation at our institution. It is possible that, with a longer period from primary surgery, there is an increased risk that patients will undergo an eventual reoperation at another clinic. However, at our institution, patients are actively followed for approximately 1 year after primary ACLR and they are given the opportunity to contact our clinic directly in the event of postoperative problems or new injuries. This reduced the influence of this limitation. The rate of graft rupture might be underestimated, as only data on patients with a confirmed graft rupture at reoperation were available. Finally, the incidence, types and risk of reoperation, relating to hardware removal in particular, are technique dependent.

## Conclusion

One sixth of all primary ACLRs underwent reoperation due to complications or new injuries within 2 years. The most common reoperations were screw removal, meniscus procedures, cyclops removal/notchplasty and reoperations due to graft rupture, including revision ACLR. Younger age (< 30 years), female gender, medial meniscus repair and lateral meniscus resection or repair at primary ACLR were associated with an increased risk of reoperation. This study provides clinicians with important data to inform patients about the short-term reoperation rates, the most common reoperation procedures and risk factors for reoperation after primary ACLR.
